# Research on fire resistance and economy of basalt fiber insulation mortar

**DOI:** 10.1038/s41598-023-44591-9

**Published:** 2023-10-12

**Authors:** Chen Ding, Kaixi Xue, Guangsheng Yi

**Affiliations:** 1 College of Civil and Transportation Engineering, Shenzhen University, Shenzhen, Guangdong China; 2 School of Civil and Architecture Engineering, East China University of Technology, Nanchang, Jiangxi China

**Keywords:** Civil engineering, Composites

## Abstract

The construction sector has become the most critical source of carbon emissions, but the existing thermal insulation materials such as thermal insulation mortar have obvious limitations, so it is urgent to develop building thermal insulation materials with superior performance and low cost. Aiming at the problem of poor bond strength of foam thermal insulation mortar, this research team selected basalt fiber as admixture to verify the influence of basalt fiber content on its performance and the economic feasibility of thermal insulation mortar. The main finding is that basalt fiber as an additive can obviously improve the crack resistance of thermal insulation mortar. When the content of basalt fiber increases from 0 to 2.5%, the compressive strength of mortar increases at first and then decreases, and the bond strength increases nonlinearly, but the thermal conductivity and dry density also increase. Therefore, the optimal content of basalt fiber is 1.5%. The improvement effect of fire resistance of thermal insulation mortar with 1.5% basalt fiber content is better. After curing for 28 days, the mass loss rate of the sample is reduced by about 11.1% after high temperature, and the relative compressive strength is increased by about 9.71% after high temperature. The raw material cost of the new fireproof thermal insulation mortar improved by basalt fiber is lower, and the cost of the finished product is reduced by 16.98%, 28.18%, 33.05% and 38.96%, respectively, compared with the four types of thermal insulation mortar already used in the market. More importantly, the economic recovery period of the new fireproof and thermal insulation mortar is undoubtedly shorter than that of alternative thermal insulation or energy storage materials, which not only achieves low emission and environmental protection, but also satisfies the economic feasibility.

## Introduction

In the context of global warming and the energy crisis, the development of high-performance and low-cost insulation mortar is of great value. At present, the main idea of developing new insulation mortar is to use admixture to improve the performance of mortar. Researchers introduce fibers, foaming agents, stabilizing agents and other new additives to improve the mechanical properties of insulation mortar and thermal conductivity, and have achieved excellent results.

Guardia et al.^1^ studied the effects of microencapsulated phase-change materials (PCM), lightweight aggregate (LWA) and cellulose fiber on the thermophysical properties of thermal insulation mortar. The results show that lightweight aggregate increases porosity and decreases strength and thermal conductivity at the same time. Cellulose fiber did not significantly change the properties of mortar. PCM not only increases the enthalpy of mortar, but also plays a filling role, and reduces the porosity and strength. However, the relationship between enthalpy and the amount of PCM is not only linear, but also depends on the composition of mortar. LWA increases the enthalpy of PCM cement lime mortar. Parracha et al.^2^ used several aggregates (expanded polystyrene, expanded cork, expanded clay and silica aerogel) to replace sand in different percentages to study the fresh and hardened state of these thermal insulation mortars, as well as their application in brick-based specimens and prototype walls, and evaluated the effect of substrate on the properties of thermal mortar. The results show that the density, mechanical properties and thermal conductivity of lightweight thermal insulation mortar are lower than that of control mortar (100% sand aggregate), and the capillary water absorption is higher. In addition, the application of mortar to the prototype wall shows enhanced mechanical properties and slightly higher thermal conductivity than that of brick-based specimens. However, the thermal conductivity of all mortars with insulating aggregates (whether used in brick substrates or prototype walls) is inferior to 0.2W/(m K), so it meets the requirements of EN 998-1 for thermal insulation mortars. Ding et al.^3^ selected silica fume as an admixture for the thermal insulation mortar test on the basis of the mortar mix ratio standard obtained by previous orthogonal tests. The results show that the silica fume mixed into the mortar has an excellent coordination effect of particle filling and hydration gel, and the mixing amount of 0 to 10% can significantly improve the bond strength and compressive strength of the mortar. The thermal conductivity and dry density of mortar are firmly identity. The dose range of silica fume increases from 0 to 4%, while the corresponding value decreases when the dose range of silica fume is from 4 to 10%. Dora et al.^4^ studied the feasibility of adding organic PCM composite to cement mortar, which was made by impregnating expanded vermiculite (EV) and decanoic acid (CA) with ethanol (EA) in vacuum. By replacing fine aggregate with different weight, PCM cement mortar mixture containing EV, CA-EA/EV-based PCM and partially adding nano-silica and coconut shell fiber combination (PSC) was prepared. The data show that the existence of PCM improves the thermal durability of the mortar board, and the addition of nano-silica and coconut shell fiber enhances the physical and mechanical strength of the mortar board.

At present, foaming agent is a common admixture used in cement-based materials. Numerous scholars will introduce independent pore structure into cement-based materials by adding foaming agents to reduce the dry density and thermal conductivity of mortar^5^. Based on the same principle, Zhang^6^, Boros^7^ and Cui^8^ significantly reduce the thermal conductivity and modify the drying shrinkage resistance of thermal insulation mortar by introducing bubble structure into the mortar. However, the introduction of independent closed-pore structure will not only reduce the thermal conductivity, but also have a negative impact on the mechanical strength, especially the bond strength of mortar matrix.

However, there are still limitations in the existing results of insulating mortar. Numerous scholars did not consider the fire resistance of insulating mortar in the selection of admixtures and introduced various organic materials, such as plant fiber^9^, polypropylene fiber^10^, polystyrene particles^11^, animal protein^12^, etc. In addition, due to the frequent occurrence of building fire accidents, the fire resistance of buildings is becoming more and more stringent^13^, but the vast majority of scientists who develop modern insulating mortars have not tested the fire resistance of insulating mortars. As organic insulation material as an external insulation layer is not heat-resistant and not fire-retardant, a building can not be ignored fire hazards, and organic materials produce smoke and toxic gases after combustion is considerably harmful^14^, to polystyrene particles as the representative of organic insulation mortar has long suffered from criticism, has been called to ban^15^. In contrast, inorganic insulating mortars are fire resistant and have a better fire prevention effect and better prospects for development. With frequent building fires in various countries and increasingly stringent fire safety standards for buildings, the study of inorganic fire-resistant insulating mortars is particularly essential.

Apart from fire resistance as a potential gap of innovation and study, and more importantly, the economic viability of the current mortar has obviously been overlooked. Some scholars selected inorganic admixtures that are quite costly, examples include aerogels^2,16^, phase change materials^1,4^, carbon fibers^17^ and various compounds^18,19^. And the costly admixture materials, although with excellent improvement effect, are also costly to acquire, which will significantly increase the project cost and cost recovery period for large-scale application on actual building facades.

In fact, at present, no scholars have tried to use basalt fiber to improve the performance of thermal insulation mortar, this direction belongs to a critical study gap. The production process of basalt fiber determines that it does not produce waste water, waste gas and solid waste in the production process, and is known as a new pollution-free green high-performance fiber material in the twenty-first century^20^. Moreover, basalt fiber is more in line with the idea of strong economic feasibility. Compared with glass fiber, the tensile strength and elastic modulus of basalt fiber are essentially the same, but its thermal stability at high temperature has obvious advantages^21^. Compared with carbon fiber, it has become a substitute for carbon fiber in numerous application fields because of its low raw material cost, simple production process and high performance-to-price ratio^21^.

Our group selected low-cost and lightweight materials in the early stage, and the optimized ratio of perlite-pyrophyllite stone powder insulating mortar was selected with the help of orthogonal tests^22^. The problem of high dry density and thermal conductivity of the mortar specimens in the optimized ratio was improved by introducing air bubbles into the slurry^23^. However, the bond strength of the foam mortar did not meet the standard of Type I insulation mortar officially required by the Chinese government, so it was necessary to continue adding new admixtures to improve the mechanical properties and test the fire resistance effect. Further scholars^6–8^ have also found the phenomenon of bond strength damage in foam mortar tests, so it is necessary to continue to adopt new admixtures to improve mechanical properties. The main objective of this paper is to select basaltic fibers as admixtures to test the improvement effect based on previous results of this group and to select the optimal ratio, with test parameters specifically related to fire resistance and economic payback period.

## Materials and methods

### Raw material selection

**Figure 1:**
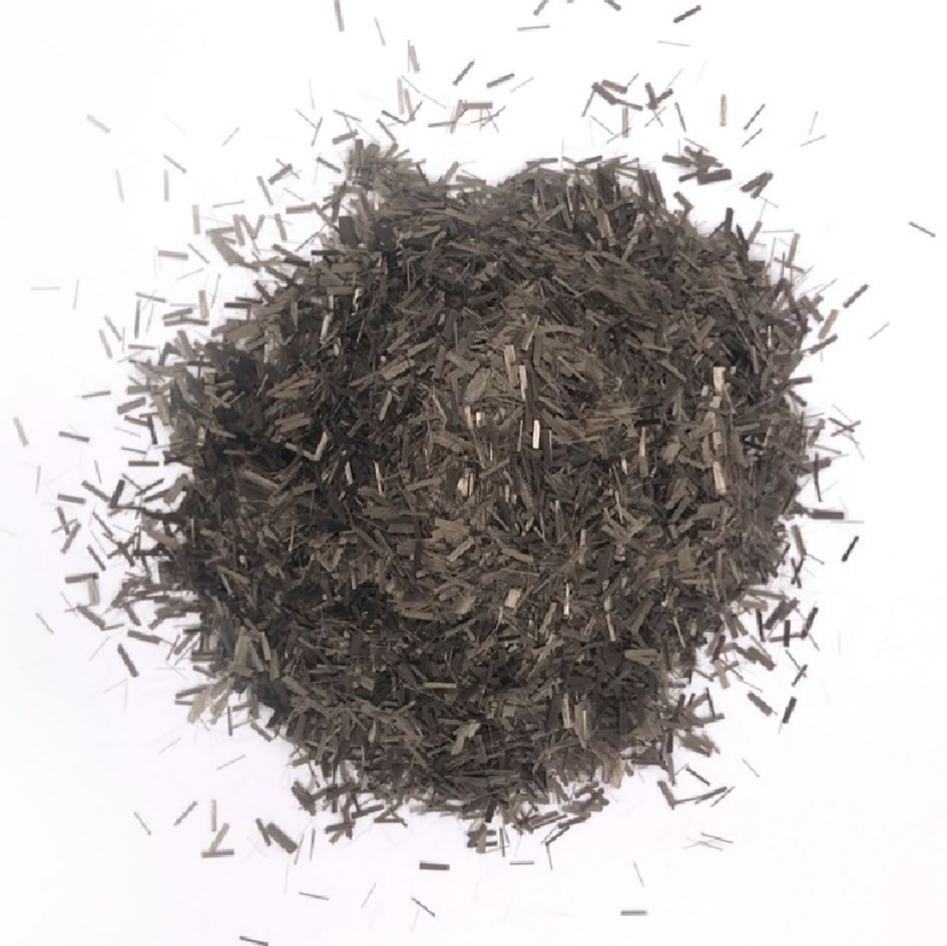
Basalt short-cut fiber (6 mm long).

In this study, basalt fiber is selected as an additive to improve the performance of mortar. Basalt fiber is an inorganic continuous fiber formed by high-speed drawing of platinum–rhodium alloy wire leakage plate after basalt stone is melted at 1450–1500 °C. The color of pure natural basalt fiber is typically brown, and the density is about 2600 kg/m^3^. Because the thermal conductivity of basalt fiber is low^22^, the high temperature stability is excellent, and the comprehensive performance is better than other fibers^24–26^, basalt fiber is selected as the admixture of thermal insulation mortar to test its improvement effect on mortar. The appearance of basalt fibers is shown in Fig. 1.

Other additives included in this paper include redispersible latex powder, fly ash, diabase powder, AOS foaming agent and gelatin. The appearance and basic information of other major materials involved in this study are shown in Table 1. The mineral types and content parameters of cement (Cement), fly ash (CFA), and diabase powder (DRP) used in this study are shown in Table 2.

**Table 1:** Basic information of other raw materials.

Materials	supplier	Basic information	Appearance picture
Expanded perlite	Hebei Yixin energy-saving insulation materials factory	This test uses expanded perlite as mortar aggregate, all the indicators meet the requirements of the specification^27–29^, large particles of lightweight aggregate closed-cell perlite, particle size 30–40 mesh or so, the bulk weight of about 70 kg/m^3^, compression ratio of about 1.5, packing density of about 100 kg/m^3^, the thermal conductivity of the test is about 0.034W / (m–k), water content of about 2%	
Flyash	Hebei Rongchang Sheng Environmental Protection Building Material Company	In this test, ultrafine grade II high quality fly ash was used, which was tested to have a thermal conductivity of about 0.3 W /(m–k) and a bulk density of about 700 kg/ m^3^. The thermal conductivity of the fly ash mineral admixture was relatively low compared to that of cement	
Diabase rock powder	Nanfeng County Zhuliang town mine	The diabase has a gray-green appearance, and the group used the ground powder of diabase as mortar externally doped with mineral powder, which was tested to have a thermal conductivity of about 0.311 W /(m–k), and a packing density of about 900 kg/m^3^. The specimen of the mother rock of diabase was in the form of gray-green lumps, which were mainly composed of plagioclase feldspar (60%), ordinary diabase (30%-35%), and a small amount of black mica (1%)	
Redispersible emulsion powder	Henan Zhengzhou Runcheng Building Materials Company	Redispersible emulsion powder (hereinafter referred to as emulsion powder), solid composition content ≥ 98.0%, packing density (g/L) is 300–500, protective colloid is polyvinyl alcohol, less than 4% of the particle size is greater than 400um, PH value 6–8. Its glass transition temperature is -10 °C, and the lowest film forming temperature is 0 °C	
AOS foaming agent	Nanchang Sasol Chemical Raw Materials Factory	Sodium α-alkenyl sulfonate (AOS) was used as a blowing agent to introduce bubbles in this experiment because the research results^30^ showed that it possesses superior foaming properties and lower cost than other types of blowing agents.AOS powder is in the form of milky white powder	
Gelatin	Nanchang Sasol Chemical Raw Materials Factory	In this test, gelatin was used as a foam stabilizer, and the combination of gelatin and blowing agent could play a very good role in reducing the bursting of bubbles^31^

**Table 2:** Chemical composition of cement, fly ash and diabase rock powder.

Raw material	Mass fraction /%								
	Al_2_O_3_	SiO_2_	Fe_2_O_3_	CaO	SO_3_	K_2_O	Na_2_O	MgO	LOI
Cement	4.05	46.20	1.81	40.35	1.56	0.66	1.02	0.64	3.71
CFA	36.96	52.96	3.65	2.12	1.05	0.88	0.22	0.73	1.43
DRP	41.40	12.71	15.66	9.19	0	0.83	6.74	13.47	0

### Sample preparation

**Figure 2:**
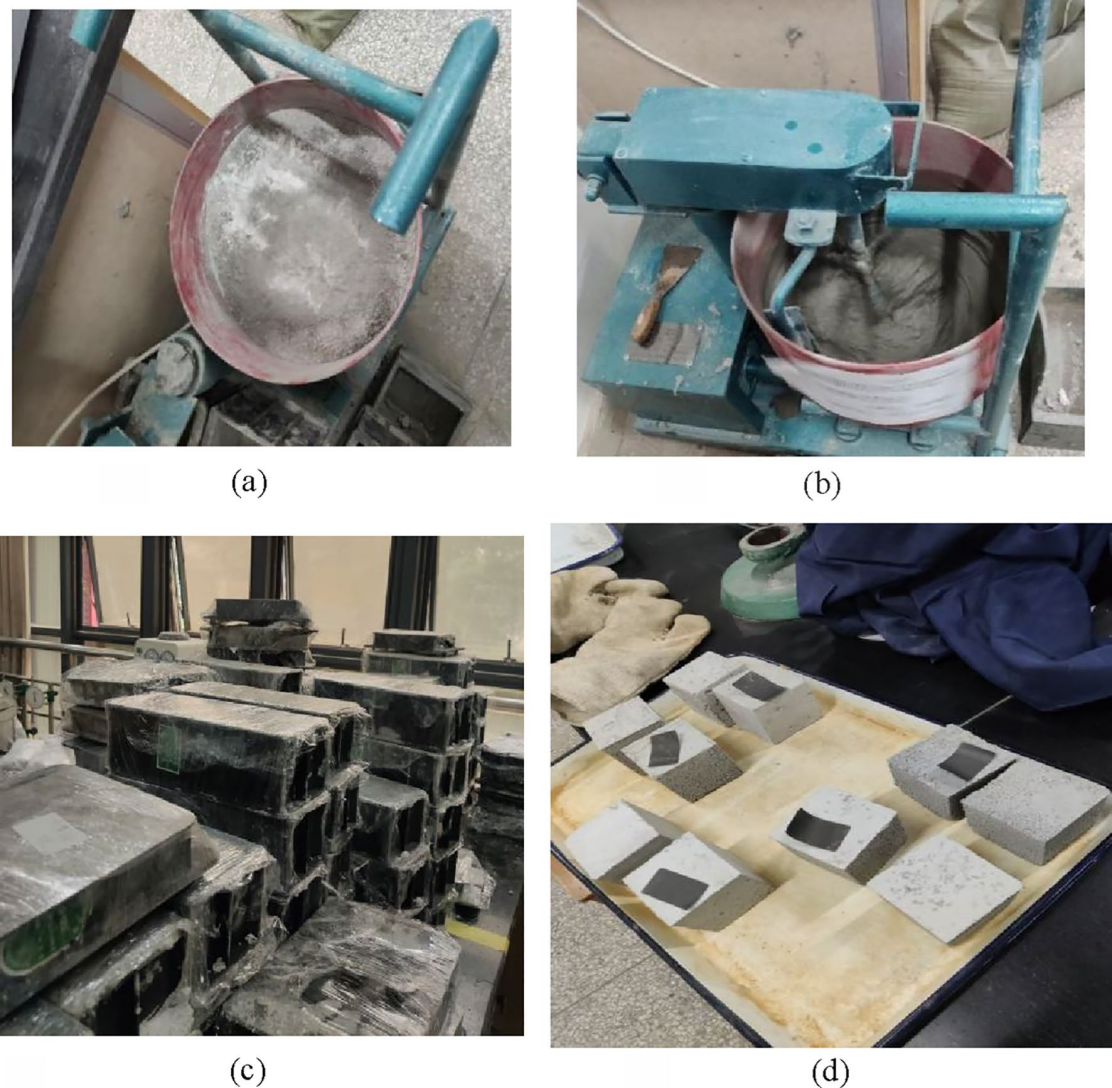
Preparation process of mortar specimen. (**a**) Mortar raw materials. (**b**) Mortar mixing process. (**c**) Mortar specimen sealing film curing. (**d**) Drying of mortar specimens.

The best dosing of each component material obtained in the improvement test phase of foam insulation mortar is used as the benchmark ratio, and the process of obtaining the benchmark ratio in the preliminary test can be referred to literature^23^, the benchmark ratio is cement: aggregate: water: redispersible emulsion powder: fly ash: diabase rock powder: AOS blowing agent: gelatin = 1:1.67:2:0.045:0.15:0.225:0.035:0.02. Referring to the relevant literature^32,33^, the dosing of basalt fibers in the modified test when fibers were mixed into the insulation mortar was set to 0.50%, 1.00%, 1.50%, 2.00%, and 2.50% in that order. Provide for a single test group admixture mixed into the mass = set admixture (expressed as a percentage) × single test group cement, mineral powder, fly ash (the main hydration cementing material) total mass, with the test group without basalt fiber as a reference group (i.e., when the fiber admixture is 0). In addition, the number of days of curing of mortar test blocks (all the days below are abbreviated as d) was taken as the test variable, and the curing times of mortar test blocks were 3d, 7d, 14d, 21d, 28d. The raw materials were mixed into the mixing barrel according to the set ratio, and after mixing, the insulation mortar would be a viscous paste, and then poured into the touching device with the corresponding test parameters, and maintained under the standard environment until the set number of days. The conditions for standard curing of insulating mortar specimens generally include two key aspects: the specimens should be maintained at a temperature of 20 ± 2 °C, and the specimens should be maintained at a relative humidity of 95% or more to prevent the specimens from losing moisture. Figure 2 show the process of mortar mixing and sample preparation.

**Figure 3:**
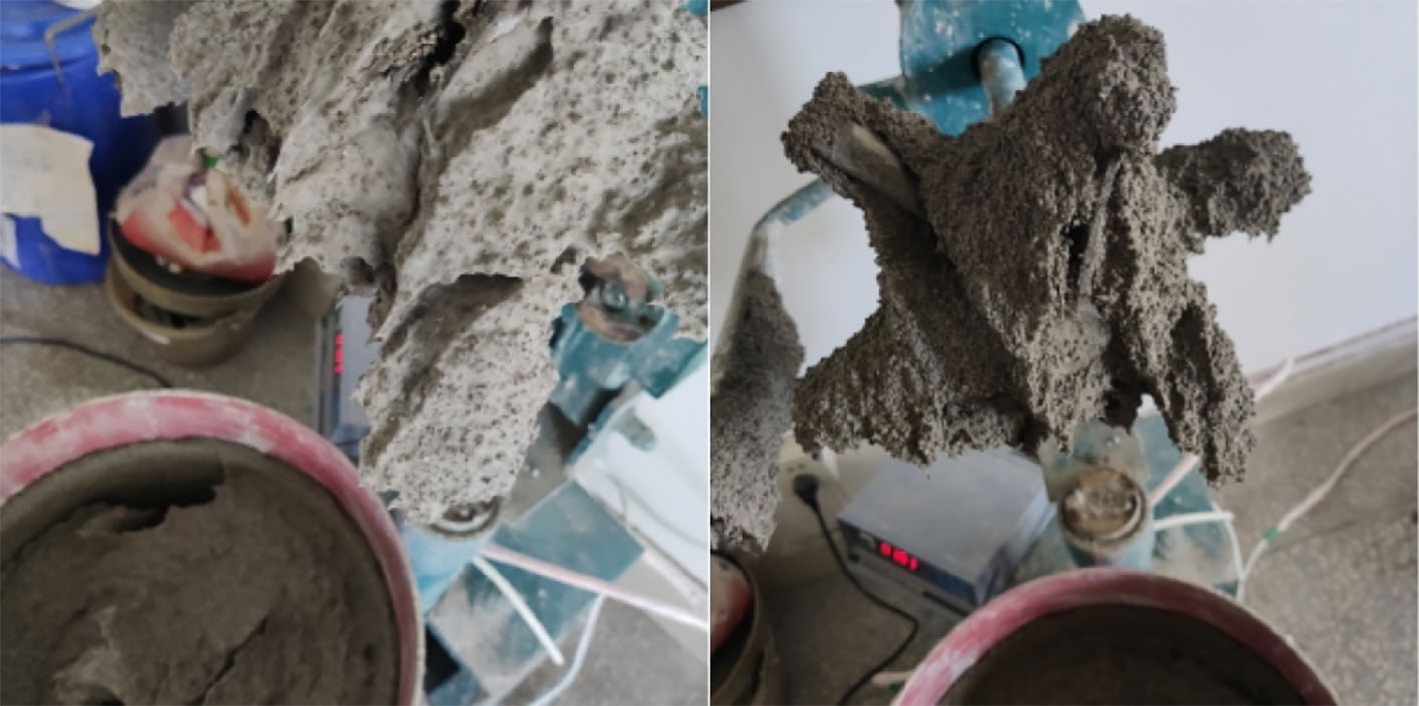
Mortar mixed with basalt fiber.

**Figure 4:**
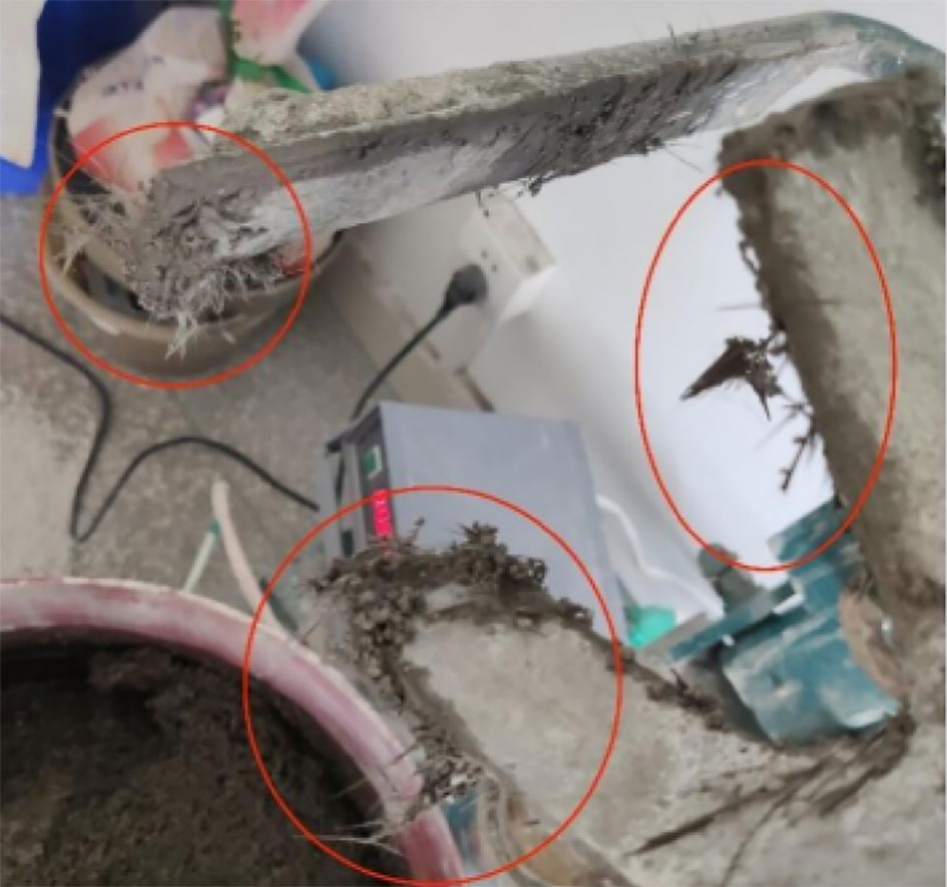
Fibers and paste adhered to the device.

In the process of mortar mixing, there are additional fibers and slurry adhering to the fan blade, and the morphology of the mortar after mixing is shown in Fig. 3. There is still a tiny amount of fiber adhering to the fan blade of the mixer after cleaning with a scraper, as shown in the red circle in Fig. 4. From the side to reflect the mortar bond performance after mixing with fiber significantly improved, but in the process of the test found that the rate of foam defoaming significantly accelerated; without adding fiber when the slurry is stationary, it takes about 15–20 min to altogether defoam, and after adding fiber the complete defoaming time is reduced to 7–9 min.

In this paper, the official Chinese specification "Building insulation mortar" (GB/T 20473-2021) is used as the standard for evaluating whether the performance of insulation mortar is excellent or not, and the specific requirements of the parameters of insulation mortar after hardening are shown in Table 3.

**Table 3:** Technical standards of building insulation mortar after hardening.

Experiment parameters	Units	Technical standard	
		I insulation mortar	II insulation mortar
Dry density	kg/m^3^	≤ 350	≤ 450
Compressive strength	Mpa	≥ 0.5	≥ 1.0
Bond strength	Mpa	≥ 0.1	≥ 0.15
Heat conductivity coefficient	W/(m K)	≤ 0.07	≤ 0.085
Linear shrinkage rate	–	≤ 0.30%	
Fire-resistant performance	–	Refer to the specification GB/T 8624-2012 in the fire A1 requirements: high temperature furnace temperature rise ≤ 50 °C, mass loss rate ≤ 50%, sustained combustion time ≤ 20 s	

### Test parameters test method

The main experimental equipment or devices involved in this paper are as follows: UJZ-15 vertical automatic 15L cement mixing testing machine; SHT4305 microcomputer-controllable electronic universal testing press; TC3000E-thermal conductivity meter produced by Xi'an Xiaxi Electronic Technology Co., Ltd.; RX3-459 large-size box-type high temperature calciner produced by Changsha far East Electric Furnace Co., Ltd. Nova NanoSEM 450field emission scanning electron microscope produced by Thermo Fisher Scientific Company (formerly FEI Company) in the United States.

**Figure 5:**
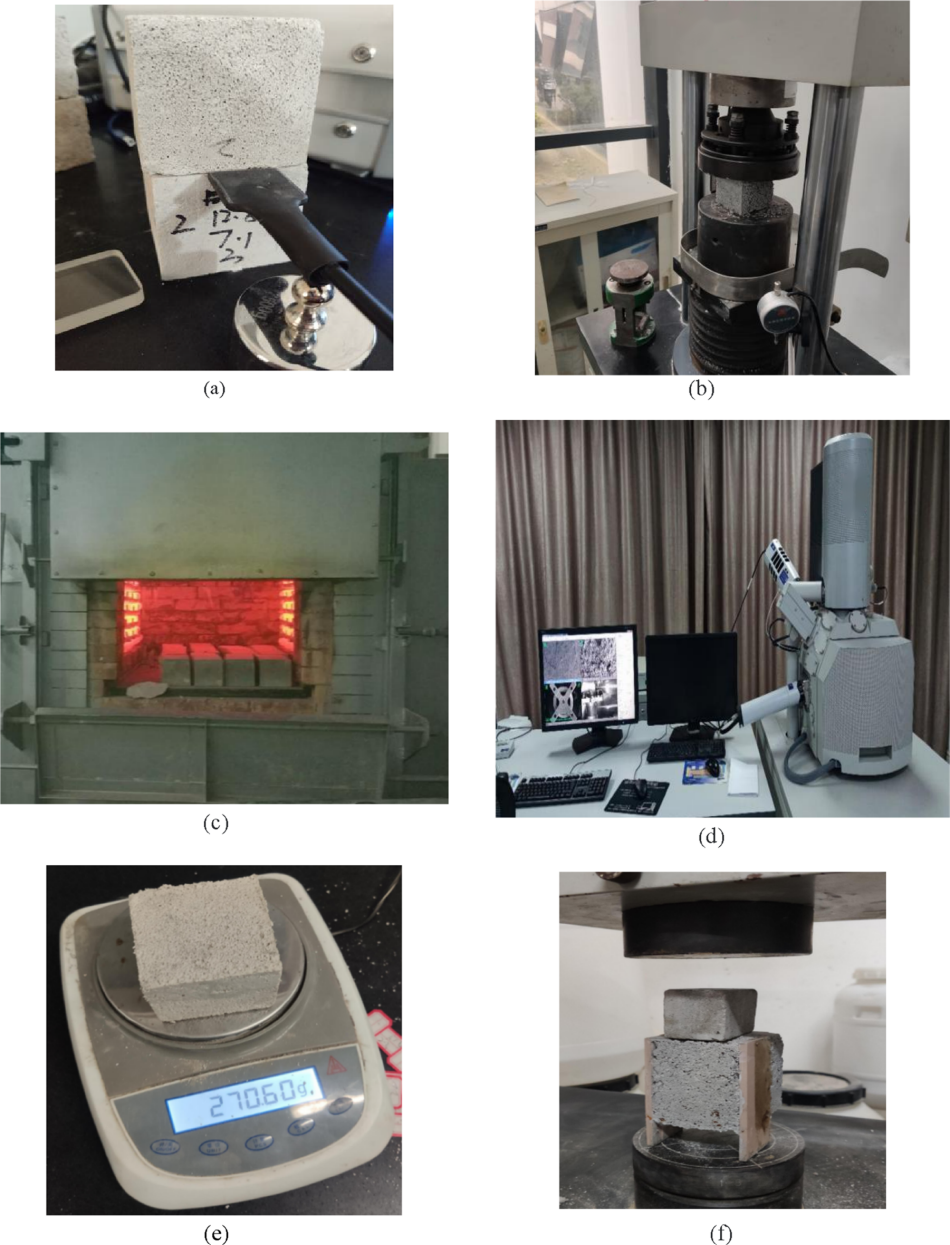
Testing process of each parameters of mortar. (**a**) Thermal conductivity. (**b**) Compressive strength. (**c**) Fire resistance. (**d**) Microstructure characterization. (**e**) Dry density. (**f**) Bond strength.

This paper takes the Chinese official code "Building Thermal Insulation mortar" (GB/T 20473-2021) as the scientific standard to evaluate whether the performance of thermal insulation mortar is excellent or not. This code is jointly formulated by many scientific research institutions and universities in China, and its scientific and authority are very guaranteed. The specific requirements of the hardened parameters of thermal insulation mortar are shown in Table 3. All test specifications and standards involved in this article, unless otherwise stated, are officially issued by China. The main test process of thermal insulation mortar test block is shown in Fig. 5. All data of multiple test results are averaged in this paper, and the test method of thermal insulation mortar is as follows.

1. Thermal conductivity: referring to "Determination of thermal conductivity and thermal diffusivity of building materials: transient plane heat source method" (GB/T 32064-2015), the thermal conductivity is measured in the thermal conductivity of the test block is measured after drying to constant weight in the oven at 105 ± 5 °C.
2. Compressive strength: according to the "Construction mortar basic performance test methods" (JGJ70-2009) in the relevant steps, including the compressive strength test need to be made into test pieces size 70.7 × 70.7 × 70.7 mm^3^ cubic block.
3. Dry density: refer to "Test Method for Inorganic Hard Insulation Products" (GB/T5486-2008).
4. Bond strength: refer to the "building insulation mortar" (GB/T 20473-2021) in the pressure shear bond strength test method, for the convenience of research on the test method has been improved, but the bond strength test principle remains unchanged.
5. Fire resistance test: referring to the method of high-temperature furnace heating to simulate fire combustion conditions recommended by the general specification "test methods for non-combustibility of building materials" (GB/T 5464-1999). The samples were calcined at high temperature using a controllable box-type resistance furnace, with one curing group for each ratio, and six test blocks were established for each curing group. The temperature in the furnace shall refer to the requirements of ISO-834 (1010 °C at 2 h), since it takes about 1 h (hereinafter referred to as h) for the equipment to warm-up to 1010 °C, the length of each group of high-temperature tests is set to 3 h. the international standard for the determination of fire resistance of building materials. Before calcination, we measured the mass m_O_ and compressive strength f_O_ of the specimen. After the combustion test for 3 h, the residual strength f_S_ and m_S_ of the sample were tested. The mass loss rate R (hereinafter referred to as burn loss ratio) was calculated according to formula (1). Calculate the relative compressive strength of mortar δ^T^ according to formula (2); the fire loss rate R after high temperature and the corresponding compressive strength δ^T^ are used as fire resistance indexes.
  1 $${\text{R}} = \frac{{{\text{m}}_{{\text{o}}} - {\text{m}}_{{\text{s}}} }}{{{\text{m}}_{{\text{o}}} }} \times 100\%$$
  2 $$\delta^{{\text{T}}} = \frac{{{\text{f}}_{{\text{s}}} }}{{{\text{f}}_{{\text{o}}} }} \times 100\%$$

(6) Microstructure observation: The scanning electron microscope (Scanning Electron Microscope, abbreviated as SEM) model is FEI Nova NanoSEM 450, and the physical object is shown in Fig. 5d. The instrument accessories consist of Inca Energy X-Max20 energy spectrometer, QUORUM-Q150R-S automatic ion sputtering coater and Gatan ChromaCL II chemiluminescent tube. The instrument extracts the air from the test chamber, and then uses the chemiluminescent tube to emit an electron beam to irradiate the gold-plated specimen, and obtains the microstructure of the specimen according to the reflection of electrons.

## Mortar performance test results

### Conventional parameter testing

**Figure 6:**
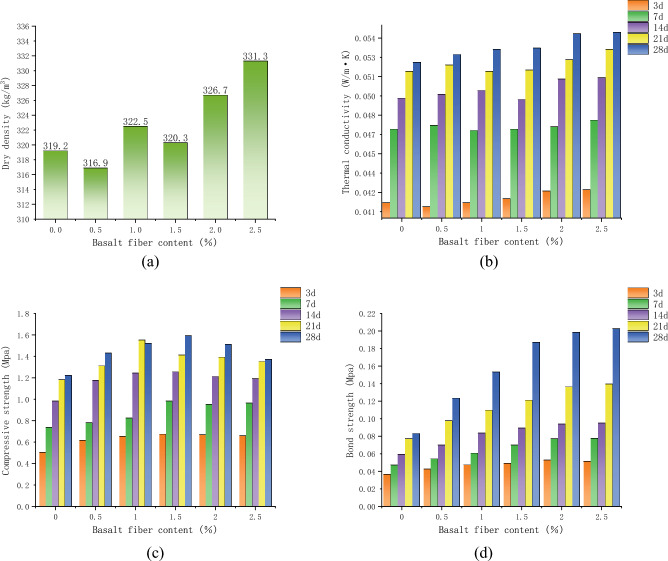
Variation of mortar properties with basalt fiber content. (**a**) Variation of dry density of mortar. (**b**) Variation in mortar thermal conductivity. (**c**) Variation in mortar compressive strength. (**d**) Variation of mortar bond strength.

In order to investigate the effect of basalt fiber on improving the thermal conductivity and mechanical properties of mortar, we conducted a two-factor experiment (including curing time and amount of admixtures). One crucial point to note is that the dry density of the mortar is not affected by the curing time, so the curing time factor was not taken into account. Experiment results for dry density, thermal conductivity, compressive strength and bond strength for each set of insulating mortar specimens as shown in Fig. 6.

There is a nonlinear growth trend between mortar dry density and fiber content. When the fiber content increases from 0 to 2.5%, the mortar dry density increases from 319.2 to 331.3 kg/m^3^, and the dry density increases by about 3.8%, which indicates that the fiber addition will increase the mortar dry density to a certain extent. The reason for this phenomenon is that in the process of sample preparation, additional bubbles break before the closed pore structure is formed in the slurry, and the dry density increases with the decrease of the closed pore structure. A tiny amount of fibers can support the bubbles and make them adhere or bond^34^, however, short cut basalt fibers are flaky or needle-like in shape, which can easily cause the bubbles to break early in the mortar mixing or other processes, and this is the reason why the complete defoaming time was greatly earlier in the test sample preparation stage. In addition, the basalt fiber material itself is exceptionally dense, at about 2600 kg/m^3^, which will increase the average density of the slurry.

Thermal conductivity did not alter significantly with the increase of basalt fiber admixture, but it still increased slightly with the increase of basalt fiber admixture, and the overall relationship between the thermal conductivity and the curing time of the mortar became positive. The compressive strength of each group of mortar showed a trend of increasing and then decreasing with the increase of basalt fiber admixture. Basalt fiber content in the range of [0, 1.5%] can improve the compressive strength of mortar, because basalt fiber has numerous advantages, such as high tensile elastic modulus, great tensile strength and excellent dispersion. After compatible combination with cement-based mortar materials, the fibers are uniformly distributed in the slurry in a disordered direction and cemented into a network space structure, which effectively improves the crack resistance, compressive strength, and integrity of mortar^35^. The 28-d mortar compressive strength reached 1.59 Mpa when the basalt fiber admixture was 1.5%, which is about 30% higher than the 28-d mortar compressive strength without fiber. When the basalt fiber admixture exceeded the critical value of 1.5%, the compressive strength of the mortar in each curing group was significantly reduced, and at this time, the fiber embodied a negative effect on the compressive strength, and the excessive basalt fiber would produce the phenomenon of staggered agglomeration, which enlarged the pore space between the aggregates and was not conducive to the formation of the internal space skeleton of the mortar^36^.

The bond strength of the mortar in all groups showed a significant increase with increasing dosage of basaltic fiber; the rate of shift of bond strength was different for mortar under different curing ages, and the bond strength of mortar in 28d curing group increased the most, and its bond strength increased from 0.083 MPa in the control group to 0.202 MPa when the basalt fiber dosage was 2.5%, and the increase in bond strength was more than 140%; basalt fiber can significantly improve the bond strength between insulating mortar and tiles. The reason for the improvement in bond strength is that the fiber, which is uniformly distributed in the slurry in a disordered direction, can form a polymerization system with the cementitious material after uniform mixing^37^, and the hydration products are adsorbed on the tile surface in concert with the fiber, The excessive amount of basalt fibers causes fiber agglomeration and the formation of internal interconnected voids, which reduces the strength-enhancing effect and leads to a gradual flattening of the bond strength enhancement curve^38,39^.

### Fire resistance test

**Figure 7:**
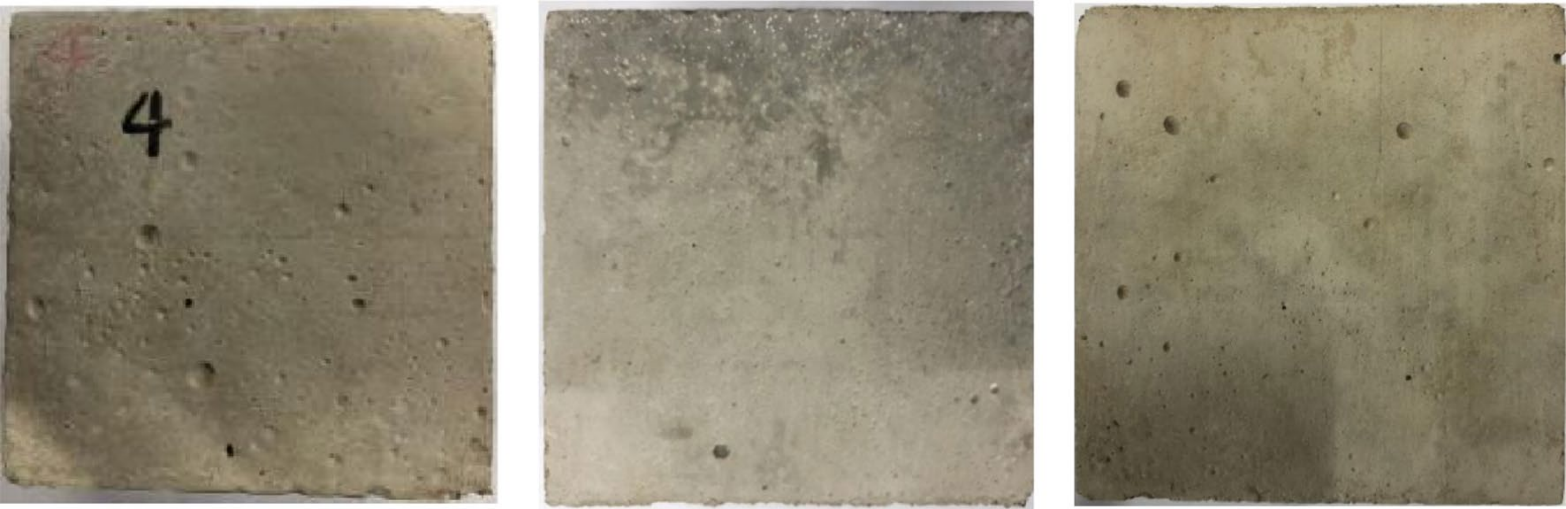
Burnt mortar test block.

The mortar specimens were placed in an elevated-temperature furnace for heating, while a temperature sensor inside the furnace was used to test the air temperature inside the furnace. The process of heating up to 1010 °C in the furnace took about 1 h, and then the constant temperature was maintained for 2 h. The total heating time for each set of mortar specimens was 3 h. During heating, physical features are evident, with the main changes being color, cracks, missing corners, and the production of white smoke. The specimens are still mostly intact, but a few have fine cracks and minute holes scattered across the surface. The surface color of the specimen adjusts with increasing temperature. In the process of temperature increasing from room temperature to 1000 °C, the surface color of some mortar specimens changed from dark gray to light gray and then to light yellow and finally to burnt black. After calcination has been completed at elevated temperatures, the surface of some specimens will exhibit a burnt black state, as shown in Fig. 7.

The temperature shift in the furnace during the whole calcination process is less than 30 °C; the mass loss of each set of mortar specimens was less than 10% and considerably less than 50% after 28 days of curing. There was never a flame burning in the furnace. According to the requirements of the Classification of Combustion Performance of Building Materials and Products (GB/T 8624-2012) (as shown in Table 3), the basalt fiber insulating mortar developed in this paper has met the standard of A1 grade of combustion performance of building materials, i.e. the most excellent grade of fire resistance.

**Figure 8:**
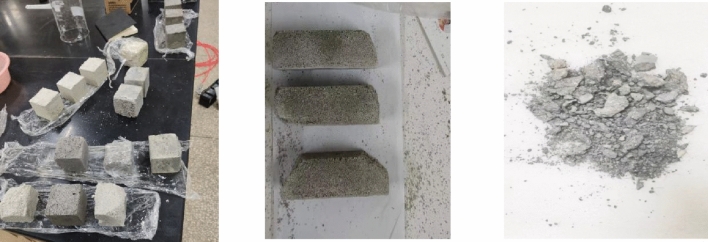
Damaged samples after high temperature calcination.

**Figure 9:**
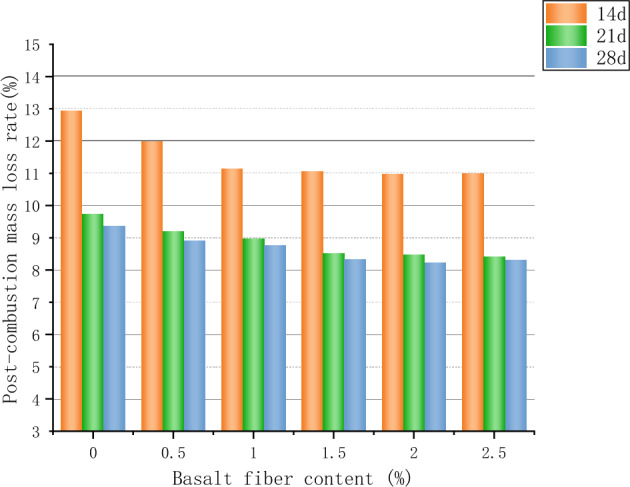
Mass loss rate of test block after high temperature.

**Figure 10:**
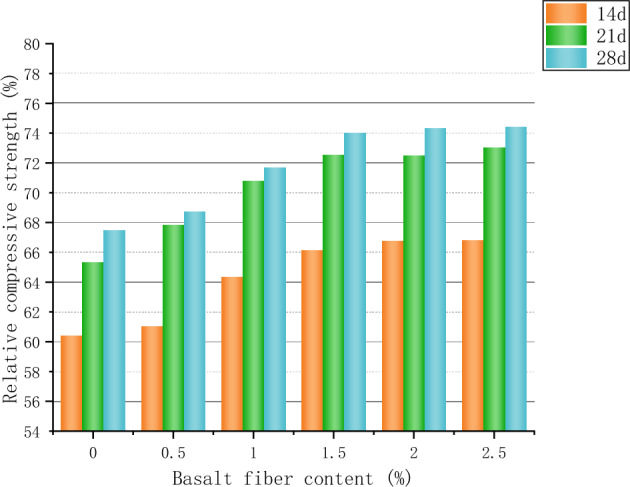
Relative compressive strength of mortar after high temperature.

After elevated-temperature calcination of the mortar, it was found that the specimens of the curing group with shorter curing time (3d and 7d) would typically show serious cracks and damage after being put into the high-temperature calciner, and some of the severely damaged specimens produced bursting, collapse and separation. The shape of the detached sample is irregular, as shown in Fig. 8, by which time the specimen has lost its condition for mechanical testing. Additional scholars have also experienced bursting when conducting mortar heat resistance tests^40^. The reason is that the curing time of the specimen is overly short, the cementitious mortar cementitious material has not been sufficiently hydrated, the bond between the coarse aggregates of the mortar has not been effectively formed, the connection between the components is looser, and then encountering the elevated temperature of 1000 °C, the decomposition of the mortar hydration products is more rapid, and the tiny cracks and pores suddenly expand can lead to the explosive splitting of the whole sample^41^. In order to more accurately explore the changes in refractory performance of different basalt fiber admixtures, the mortar curing time during the refractory test was not considered for 3d and 7d specimens, and the tests were conducted for 14d, 21d, and 28d. The burn loss rate and relative compressive strength of the mortar after elevated temperature calcination are shown in Figs. 9 and 10, respectively.

In the course of calcination, complex physical and chemical changes occur in the interior of the test mass, and all the free water is evaporated, and the combined water is gradually dissipated, leading to a loss of its mass. When the fiber dose was increased from 0 to 2.5% and the mortar burning loss rate decreased in both the 21d and 28d curing groups, the mortar burning loss rate decreased by about 13.55% in the 21d curing group and the mortar mass loss rate decreased by about 12.25% in the 28d curing group. The decrease of mass loss rate after high temperature with the increase of basalt fiber admixture is due to the good fixing and reinforcing effect of fiber, and the excellent high temperature resistance of basalt fiber itself^42^, and the cohesive force formed by expanded perlite aggregate, cementing material and fiber together after fiber admixture in mortar can significantly reduce the cracking, spalling and even explosive separation due to fiber admixture^41^. The quality loss rate of the mortar in the 14-day curing group remained essentially above 10% after the addition of fibers because the curing time was not prolonged enough, the internal hydration products of the mortar had not yet reacted thoroughly, and the effect of basalt fibers and cementation and fixation had not been sufficiently reflected. Mortar curing times increased from 14 to 28d for all groups, corresponding to a decreasing trend in the mass-loss rate, but the data difference between mortar curing times for 21d and 28d groups was smaller.

Basalt fibers have excellent refractory and high temperature resistance^43^, and can give full play to the high tensile modulus and tensile strength in cementitious materials^44^. According to the shift of relative compressive strength, basalt fiber has a positive effect on the residual strength of insulating mortar, when the basalt fiber admixture is from 0 to 2.5%, the relative compressive strength of mortar in each curing group at 14d, 21d, and 28d is increased by 10.56%, 11.75%, and 10.29%, respectively, and the rate of increase is slowed down after the basalt fiber admixture is greater than 1.5%. The increase in the residual strength of cementitious materials after elevated-temperature calcination of fiber-doped mortars has been confirmed in numerous academic papers^40–42,45,46^, due to the spatial system formed by the disordered distribution of the uniformly distributed and compatible combination with cementitious mortar materials, which acts as a "support" for the expanded perlite aggregates, and the fibers between the microcracks act as a "bridge" to improve the integrity and cohesion of the whole specimen, thus reducing the generation of cracks during calcination. The interfacial adsorption of adhesion between the mortar matrix and fibers allows the internal cementation of the mortar into a network space structure, which can also contribute to the residual compressive strength^41,46^. The relative compressive strength of the mortars in all curing groups increased with the number of curing days, indicating that the continuous progress of the cement hydration reaction has a positive effect on the refractory properties of cemented mortars.

### Microscopic mechanism analysis

Basalt fiber has strong chemical stability, while cement-based materials are weakly alkaline, thus basalt fiber will not participate in the hydration reaction of cement-based materials^47^, nor will it shift the types of hydration products of mortar^48^.

The introduction of fibers into an insulating mortar affects the microstructure of the mortar to a certain extent, which affects its overall performance. Based on the test data of the mortar parameters, it can be seen that by increasing the amount of basalt fiber, the insulation mortar bond strength is significantly increased, the compressive strength is first increased and then decreased, the mortar fire resistance is also improved, while the thermal conductivity and dry density of the mortar are slightly increased. Data shift laws indicate that the bond strength enhancement is most pronounced in insulating mortars. As the material composition of cement hydrate has not changed, it is necessary to test the microstructure of basalt fiber thermal insulation mortar in order to better explore the strength improvement mechanism of basalt fiber thermal insulation mortar.

**Figure 11:**
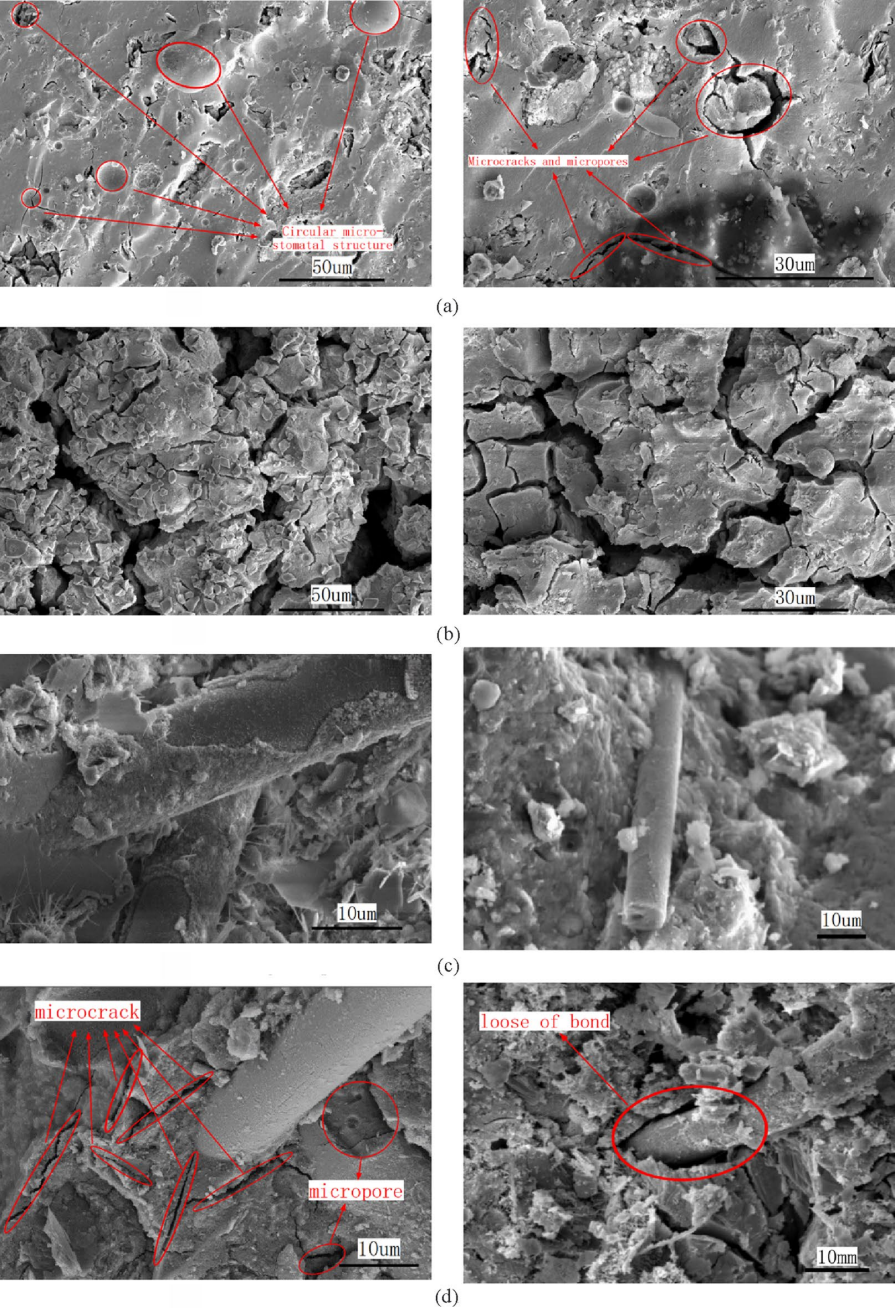
Electron microscope scanning images of four groups of samples. (**a**) Before high-temperature calcination of mortar in the control group. (**b**) After high-temperature calcination of mortar in the control group. (**c**) Before high-temperature calcination of basalt fiber insulation mortar. (**d**) After high-temperature calcination of basalt fiber insulation mortar.

The mortar samples before and after elevated-temperature calcination were selected for microscopic tests to compare the changes in the microscopic morphology of the cement matrix before and after the high-temperature tests for basalt fiber insulation mortar dosed with 1% (hereinafter referred to as the optimization group). Meanwhile, in order to better characterize the utility of basalt fiber in mortar, we also set two groups (before and after extreme temperature calcination) of unadulterated fiber insulation mortar samples as a control group. The microscopic morphology of the specimens are shown in Fig. 11a–d.

According to the microstructural images of the mortars scanned by electron microscopy, the control group of mortars differed significantly before and after calcination. Before heating, the hydration products of the control samples were dense and monolithic, and at that time, microcracks and microporosity existed in the samples, but were fewer numerous. However, after high-temperature heating, the size and number of micropores and microcracks of the samples increased significantly, which in turn negatively affected the mechanical properties of the mortar. The samples in the optimization group were tightly bonded to the mortar matrix prior to calcination at high temperature, and the hydration product matrix tightly wrapped the basalt fibers, resulting in a large bond and gripping force, which gave full play to the excellent tensile strength of the basalt fibers and enhanced the tensile crack damage resistance of the weak interfaces of the mortar matrix. After the optimization group was subjected to high-temperature calcination at 1000 °C, the mortar was internally damaged and the compaction deteriorated, causing the voids and cracks to increase, and the microvoids led to the weakening of the grip and bond between the basalt fibers and the hydration product matrix, which in turn weakened the mechanical properties. However, although the structure was loosened and damaged after high temperature calcination, the fibers could still effectively inhibit the development of microcracks, and the optimized group of specimens could still remain in relatively excellent condition, and the specimens as a whole did not crack or collapse, and the residual strength after high temperature was considerably improved compared to that of the mortar without fibers.

After microscopic analysis, it can be concluded that the fiber insulating mortar, after high temperature calcination, undergoes a series of physicochemical changes due to moisture removal or thermal decomposition of certain physical phases, causing the initiation and expansion of microcracks and micropores in the slurry, which in turn leads to a partial loss of mass of the test block and a reduction in compressive strength.

With reference to other test results of basalt fibers^49–51^, it can be seen that the inclusion of basalt fibers not only effectively resisted the mortar structural cracking, but also improved the integrity and densification of the sample. The hydration products in the mortar have increased resistance to decomposition at high temperatures, and the test blocks are fewer prone to spalling, cracking and loosening, thus reducing the rate of mass loss. The high melting point of basalt fiber, its excellent bond and bridging and crack-blocking effects with cement mortar limit the volume change of the mortar after high temperature, reduce the initiation and expansion of microcracks and micropores in the mortar, and to some extent have a moderating effect on the high temperature deterioration of the mortar, which can effectively improve the residual strength of the mortar after high temperature, and finally improve the fire resistance of the mortar.

### Optimum basalt fiber content

Basalt fiber has achieved excellent results in the improvement of expanded perlite thermal insulation mortar. With the increase of dosage, the bond strength of thermal insulation mortar increases, while the compressive strength and fire resistance first increase and then decrease. Although the thermal conductivity and dry density are slightly improved, they still meet the standard of type I thermal insulation mortar. The parameter standards of thermal insulation mortar in the latest specification^29^ are shown in Table 3. As an inorganic admixture, the cost of basalt fiber is lower than that of other component materials. Considering the cost factors, fire resistance and compressive strength changes, 1.5% is selected as the optimal dosage of basalt fiber.

## Economic analysis

### Material cost comparison

**Figure 12:**
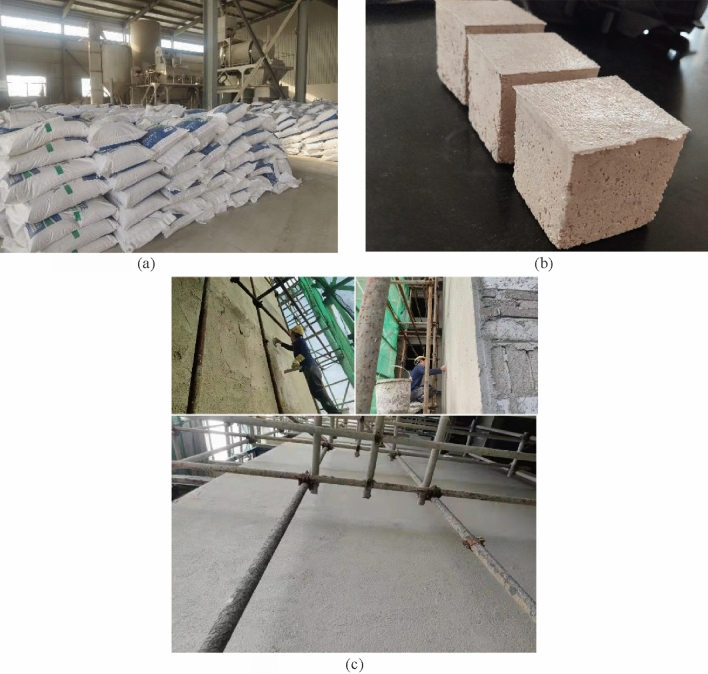
Cost control mortar. (**a**) Dry powder of insulation mortar. (**b**) Mortar Control sample. (**c**) Mortar construction pictures.

After the optimal ratio of thermal insulation mortar is obtained, the cost is compared with that of thermal insulation mortar commonly used in the market. First investigate the purchase cost of each component material, then calculate the price per cubic meter of the mortar dry powder mixture. The cooperative manufacturer of our research group is Nanchang Yintai Building Materials Co., Ltd., The finished thermal insulation mortar produced by this company has been used in the insulation layer of numerous residential buildings such as Shuanggang Garden Placement House in the Nanchang area, as shown in Fig. 12. Through the visit and investigation, the proportions of four types of mortar products are obtained, which are named DZ-01, DZ-02, DZ-03 and DZ-04, respectively, in which the DZ-04 group is the best construction quality (the highest level) of Yintai Building Materials Company, and serves as the control group in the cost analysis. The mortar ratio for each cost control group is shown in Table 4. After the basalt fiber is optimized, the test group is named XW. It should be noted that the cost described in this paper only considers the purchase cost of mortar raw materials, and additional expenses, such as transportation costs, human materials and machinery costs during construction, building operation energy consumption costs and repair costs after delivery are too floating, are not included in the scope of this calculation.

**Table 4:** Mortar mix ratio of cost control group.

Control group mortar number	Glass beads	ordinary portland cement	Redispersible latex powder	Polypropylene mesh fiber
DZ-01	1	1	0.04	0.025
DZ-02	1	1	0.12	0.020
DZ-03	1	1	0.16	0.015
DZ-04	1	1	0.2	0.010

As can be seen from Table 4, the cost control group mortar uses vitrified beads as aggregate, and the selected fiber is polypropylene reticular fiber (hereinafter referred to as fiber). Unless otherwise stated, all settlement prices involved in this article are in Chinese currency (RMB). Our research group investigated the Internet Mall in China and found that the market prices of various raw materials are shown in Table 5. Since diabase rock powder is currently a mining waste stone, the diabase rock powder used in this test was given by the manufacturer and therefore the price was set at 0 yuan. The quota valuation method is commonly adopted in the actual construction process, that is, the plastering mortar of the thermal insulation layer of the external wall represents the plastering workload by unit volume (m^3^), and then calculates the project price by the plastering workload × construction cost quota.

**Table 5:** Test raw material market price.

Raw materials	Market price per ton of raw material (yuan)
Expanded perlite	1450
Glass beads	1660
Ordinary silicate cement	680
Redispersible latex powder	17,200
Diabase rock powder	0
Fly ash	390
AOS foaming agent	14,600
Gelatin foam stabilizer	14,000
Basalt fiber	12,800
Silica fume	700
Polypropylene mesh fiber	14,600

With reference to the idea of quota valuation in the project cost, this paper directly compares the total raw material cost of preparing 1m^3^ volume dry mixed products as the raw material cost of mortar, and estimates the weight and cost of each material per ton of mortar according to the proportion of each group of mortar, as shown in Table 6. The cost of each material in the finished mortar per ton in the table = the proportion of component materials to the mortar mixture × the market price, the mortar price per unit volume in the table = the sum of the cost of each material in the dry mixture of 1 ton mortar × the dry mixture weight per unit volume of mortar (bulk density). The mortar density data (kg/m^3^) in the table are provided by the cooperative manufacturer, and the dry mixture weight per unit volume of mortar (kg/m^3^) of XW mortar is measured by the laboratory.

**Table 6:** Cost calculation results of mortar for each group.

Test group number	Raw materials	Mortar ratio	Mass of each material per ton of mortar (kg)	Cost of each material per ton of mortar (yuan)	Mass of dry mixture per unit volume of mortar (kg/m^3^)	Cost per unit volume of mortar dry mix (yuan)
XW-01	Expanded perlite	1.67	527.65	765.09	260	409.21
	Ordinary silicate cement	1	315.96	214.85		
	Emulsion powder	0.045	14.22	244.58		
	Diabase rock powder	0.225	71.09	0		
	Fly ash	0.15	47.39	18.48		
	AOS foaming agent	0.035	11.06	161.48		
	Gelatin foam stabilizer	0.02	6.32	88.48		
	Basalt fiber	0.02	6.32	80.90		
	Total	3.165	1000	1573.86		
DZ-01	Glass beads	1	484.26	803.87	300	492.93
	Cement	1	484.26	329.29		
	Emulsion powder	0.04	19.37	333.17		
	Fiber	0.025	12.1	176. 76		
	Total	2.065	1000.0	1643.09		
DZ-02	Glass beads	1	467.3	775.70	260	570.54
	Cement	1	467.3	317.76		
	Emulsion powder	0.12	56.07	964.49		
	Fiber	0.020	9.35	136.45		
	Total	2.14	1000.0	2194.4		
DZ-03	Glass beads	1	459.8	766.22	250	611.21
	Cement	1	459.8	312.64		
	Emulsion powder	0.16	73.6	1265.28		
	Fiber	0.015	6.9	100.69		
	Total	2.175	1000.0	2444.83		
DZ-04	Glass beads	1	452.5	751.15	250	670.39
	Cement	1	452.5	307.7		
	Emulsion powder	0.20	90.5	1556.6		
	Fiber	0.010	4.5	66.1		
	Total	2.21	1000.0	2681.55		

Compared to the mortar cost data for each group, the dry mix cost per unit volume of mortar in the XW group decreased by 16.98%, 28.18%, 33.05% and 38.96%, respectively, compared to the cost control group. It is known that the thermal insulating mortar prepared by the XW group meets standard performance requirements while being fewer costly than commonly used thermal insulating mortars in the market.

### Economic payback period analysis

After obtaining the optimal ratio of insulation mortar, the impact of various insulation materials on the energy consumption of typical multi-story office building air conditioning was simulated using DesignBuilder 6.0 software. Based on the simulation of building energy consumption results, the economic effect of applying the optimal group of insulation mortar was evaluated. In the analysis of engineering economic payback period, there are two indicators: static payback period (SPP) and dynamic payback period (DPP). Numerous scholars use SPP for economic analysis^52–55^, and the SPP calculation formula is shown in Eq. (3); however, this method does not take into account the time value of currency, and the payback period of insulation materials is longer, resulting in inaccurate calculation of investment payback period. Therefore, in this study, we will consider the time value of currency and conduct economic analysis on the application of thermal insulation material (TIM) using both SPP and DPP. The dynamic payback period refers to the time period required to recover all investments when considering the time value of currency. The time value of currency is included by calculating the annual net annual income based on the discount rate. The DPP after TIM application is shown in Eq. (4).

3 $$\frac{{C_{TIM} }}{S} = SPP$$

4 $$\sum\limits_{{{\text{t}} = 0}}^{{{\text{DPP}}}} {{\text{S}}(1 + {\text{r}})^{{ - {\text{t}}}} - {\text{C}}_{{{\text{TIM}}}} = 0}$$

where SPP is a static payback period, DPP is dynamic payback period, r is the discount rate, S is the annual income generated from energy savings, C_TIM_ is the investment required to purchase and install TIMs.

Considering the time value of the currency, the calculated payback period is typically larger than the value calculated by SPP; in fact, DPP is closer to real energy management and is therefore widely used for technical and economic analysis purposes. According to a report released by the National Bureau of Statistics of the People's Republic of China, energy prices are rising by an average of 4.01% (1998–2014) per year, and the long-term trend of rising energy prices is expected to continue in the coming decades^56^. In fact, with the same amount of energy savings per year, the upward trend in energy prices will make a beneficial contribution to the economic benefits of TIM applications in buildings. To ensure more reliable results, this study assumes that energy prices remain constant, it means that if the economic analysis of the TIM application is positive, then the increase in energy prices will lead to additional energy-saving profits in the actual project.

**Figure 13:**
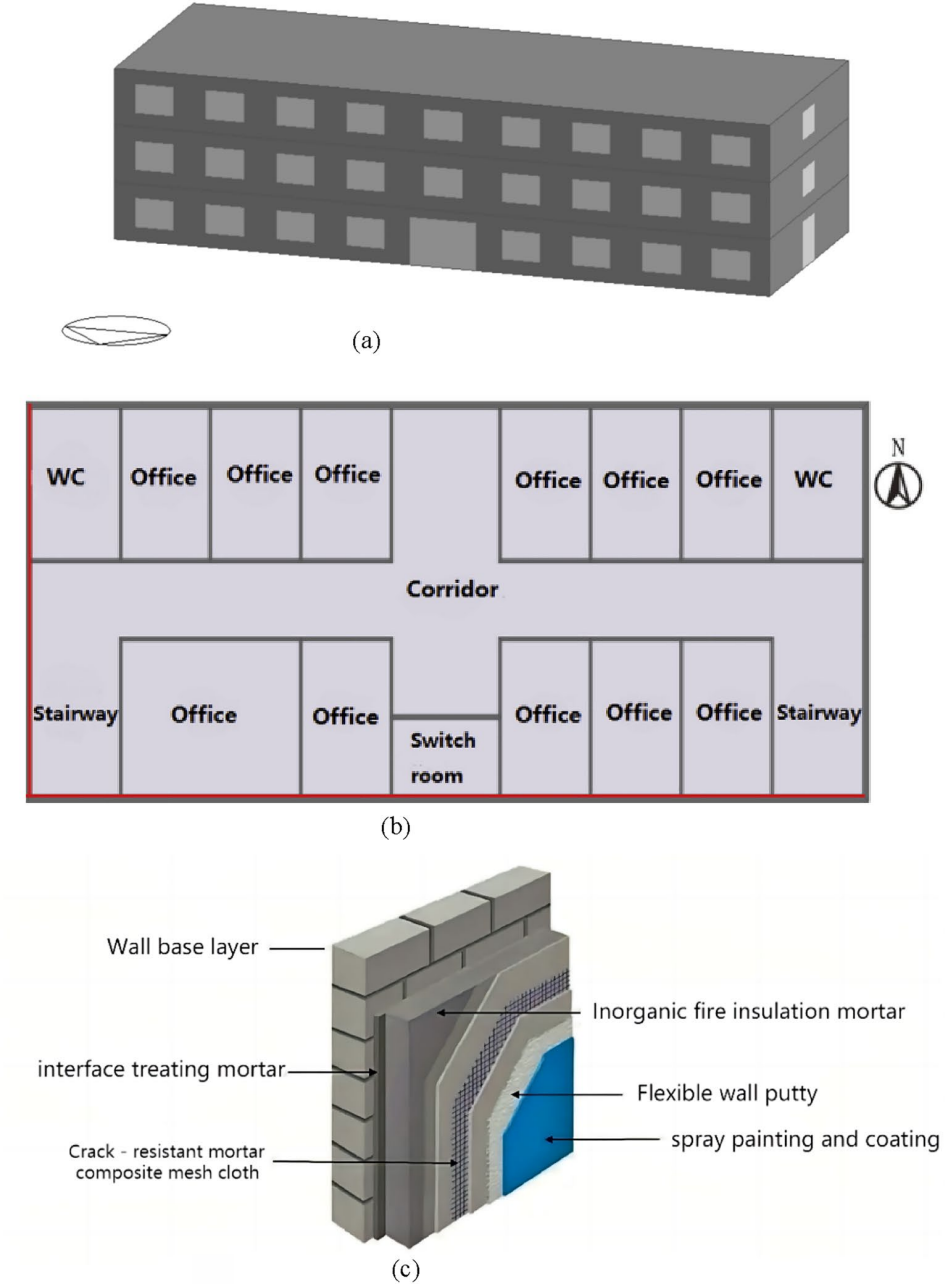
Typical three-story office building model. (**a**) Architectural models. (**b**) First floor plan. (**c**) Typical inorganic insulation mortar system structure.

In this paper, a typical multi-story office building is selected for simulation, as shown in Fig. 13a. Since other building energy consumption cannot be changed by exterior wall insulation, this paper therefore only calculates the energy consumption generated by the air conditioning and cooling system for one month during the high temperature period in summer, and then extrapolates the annual air conditioning electricity cost based on the energy consumption of individual months. The building has three floors, each floor is 3.3 m high, and the construction area of a single floor is 646.58 square meters (41.85 × 15.45 m). The size of each window is 2.4 m × 1.8 m, and the distance between the bottom of each window and the floor is 0.9 m. The ratio of window wall area on the south and north sides is fundamentally the same, both being 18%, and the building exterior wall area is 1506.64 m^2^, which is within the range recommended by the thermal design code for civil buildings (GB/T 50176-1993). The solar transmittance is set to 0.45, and the red line of the external wall in Fig. 13b is the area of direct sunlight. The thickness of window glass is 3 mm, and the visible light transmittance and thermal conductivity of the glass are 0.7 and 0.9 W/m K, respectively, depending on the magnitude of solar radiation. insulation materials are arranged on the building envelope (including the roof), and the model facade is referenced to the typical structure of inorganic insulation mortar, as shown in Fig. 13c. The main details of the building envelope structure and the thermophysical properties of the building materials are shown in Table 7, respectively, where the thermophysical parameters of the insulation depend on the different materials.

**Table 7:** The main parameters of architectural model analysis.

		Thickness (mm)	Thermal conductivity (W/m k)	Specific heat (J/kg k)	Density (kg/m^3^)
Exterior walls (from outside to inside)	Cement mortor	5	0.93	1050	1800
	Reinforced conctete	200	1.74	1380	2500
	insulation layer	50	…	…	…
	Cement mortor	12	0.93	1050	1800
Roof (from outside to inside)	Cement mortor	5	0.93	1050	1800
	Reinforced conctete	40	1.74	1380	2500
	insulation layer	25	…	…	…
	Reinforced conctete	50	1.74	1380	2500
	Cement mortor	10	0.93	1050	1800
Floor (from outside to inside)	Cement mortor	5	0.93	1050	1800
	insulation layer	25	…	…	…
	Reinforced conctete	120	1.74	1380	2500
	Cement mortor	5	0.93	1050	1800
Heating ventilation air conditioning	Heating setpoint temperature	20 °C			
	Equipment	Boiler			
	Cop	0.78			
	Cooling setpoint temperature	26 °C			
	Equipment	Air conditioning system			
	Cop	3.28			

The temperature conditions outside the building model refer to the meteorological data of Shenzhen City in July 2022 (from the official website of Shenzhen Meteorological Bureau). Five kinds of external wall thermal insulation conditions are selected for simulation, which are control group thermal insulation mortars without foaming agent and basalt fiber (details of control mortar can be found in literature^22^, named JZJ) and finished products obtained by Yintai Building Materials Company (DZ-04 group mortar mentioned in the previous section, named JYT), foam mortar without basalt fiber (details can be found in reference^23^, named JFP), modified thermal insulation mortar with 1.5% basalt fiber content (named JXW), working condition without thermal insulation measures (named WBW).

The relevant thermophysical parameters of each thermal insulation material are shown in Table 8. The average temperature of the interior surface of the building walls and the energy consumption of the air-conditioning system were calculated using the uninsulated working conditions as a control group.

**Table 8:** Thermal physical parameters of thermal insulation materials.

	Thermal conductivity (W/m k)	Specific heat (J/kg k)	Density (kg/m^3^)
JZJ	0.0637	150	414
JYT	0.0915	170	430
JFP	0.0514	140	310
JXW	0.0528	200	320

In this study, the thermal comfort temperature range was set to 20–26 °C, i.e., the heating system worked naturally when the indoor temperature was lower than 20 °C, and the cooling system ran when the indoor temperature exceeded 26 °C. DesignBuilder had an air conditioning system in the whole building model, and the cooling system was set to stay on for 24 h, and the air conditioning system started to run automatically when the temperature was higher than 26 °C or lower than 20 °C. The temperature is recorded every hour, and the model starts running from 0:00 on July 1 and stops at 24:00 on July 31.

**Figure 14:**
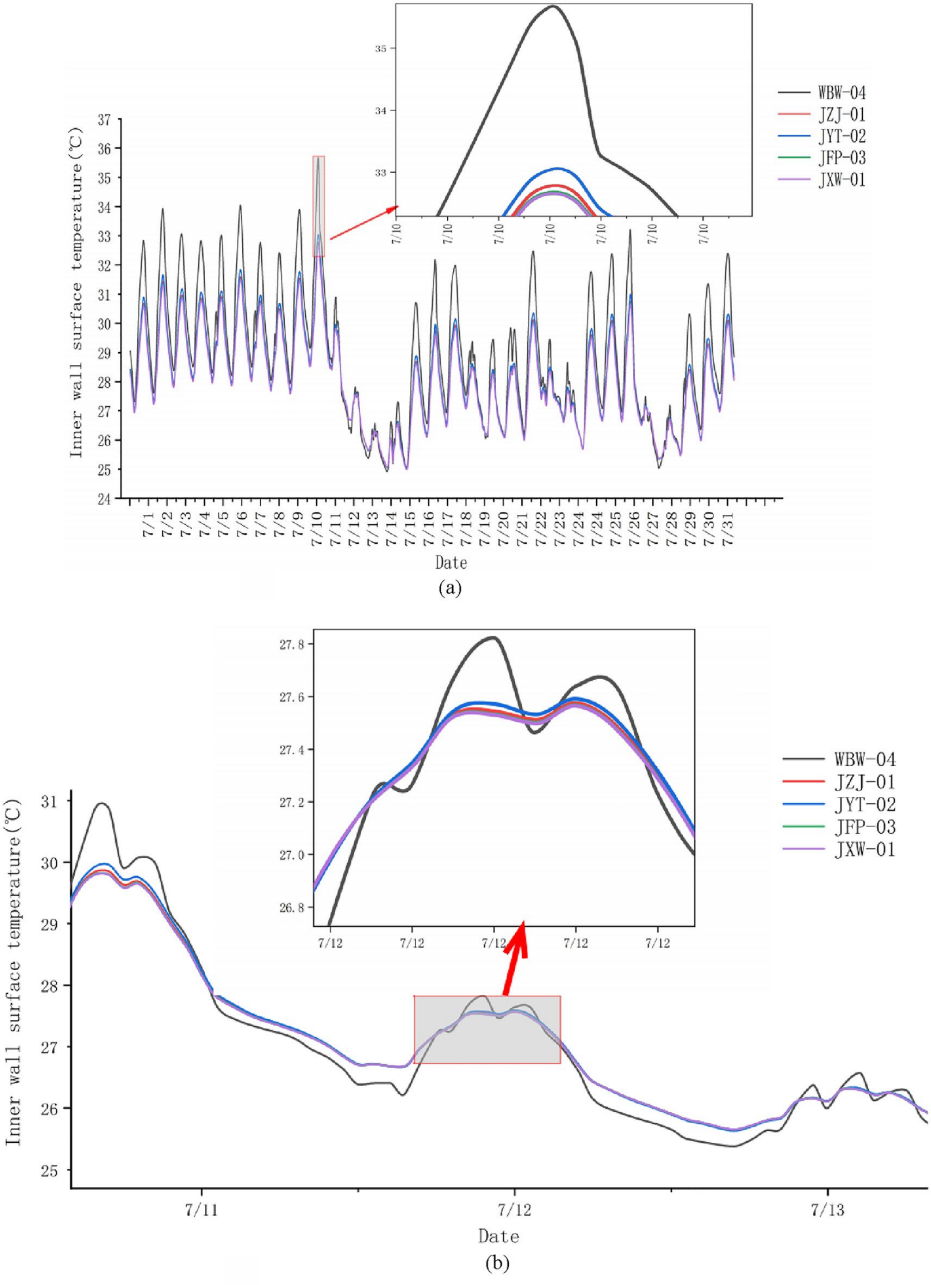
Variation of the mean temperature of the inner surface of the wall in July. (**a**) Temperature variation and local detail enlargement. (**b**) Zoomed-in view of temperature change around July 12.

The average temperature changes of the inner surface of the building model wall (external wall) under high temperature and the thermal insulation conditions of each group in July summer are shown in Fig. 14. The highest temperature of the inner surface of the wall occurs between July 10 and July 11, 2022. Due to the large amount of data, it is not easy to observe the temperature shift pattern, so Fig. 14a shows a detailed enlargement of the temperature graph (the part delineated by the red line box) for this time period.

Because there are no heat preservation measures in the WBW group, the temperature fluctuation is the largest. Figure 14b zooms in on the time period (around July 12) when the WBW group's operating temperature fluctuation shift are more pronounced. The energy consumption of the air conditioning system of a typical building model for the whole month of July is shown in Table 9. It is known that during daylight period, building insulation can prevent part of the heat from entering the indoor area, while at night (during the period without daylight), building insulation can reduce the indoor heat flow loss, and the more effective the insulation is in regulating the effect of indoor living comfort and reducing energy consumption in the area where the temperature difference between day and night is larger.

**Table 9:** Air conditioning energy consumption of buildings in July under the action of heat insulation of each insulation layer.

Insulation material types	Energy consumption (kW h)
WBW	9689.296
JZJ	5316.556
JYT	5696.492
JFP	5162.743
JXW	5123.666

According to Fig. 14 and Table 9, it can be seen that: the temperature fluctuation of the inside wall of the building under five different types of insulation wrapping is large, but the model without insulation measures (WBW group) has the most obvious temperature shift, and the temperature increase and decrease are caused by daytime heating and nighttime cooling, respectively. The best thermal insulation and heat rejection effect among the five temperature working conditions is the mortar of the JXW group, and also its energy consumption. The mortar of the JXW group has the lowest energy consumption, the lowest temperature change, and the most beneficial to indoor human air comfort. The temperature change of the control group is most obvious due to the high thermal conductivity of the wall, and the peak temperature of the control group is the highest when the outside sunlight warms up in summer, and the temperature decreases the most when the outside world cools down, and the corresponding energy consumption of the air conditioner is also the highest. JZJ, JYT, JFP and JXW reduce energy consumption by 45.13%, 41.21%, 46.72% and 47.12%, respectively, compared with WBW without insulation measures. The JXW group has the most excellent effect of energy saving and emission reduction.

After inquiring that the electricity price of ordinary residents in Shenzhen in the summer of 2022 is 0.95 yuan per kilowatt-hour (0.95 RMB/kW h), the whole building is regarded as a complete unit. Compared with WBW without insulation measures, JZJ, JYT, JFP and JXW insulation could save 4154.10, 3791.16, 4300.23 and 4337.35 yuan in electricity costs in July, respectively. Since the energy consumption of buildings is known to vary from month to month, this paper estimates the energy consumption of the air conditioning system for the whole year based on the ratio of the electricity consumption in Shenzhen in July to the total electricity consumption for the whole year..Since the electricity consumption of Shenzhen for the whole year and July has not been announced, this paper estimates that the annual electricity consumption of Shenzhen will reach 110.34 billion kW h in 2021 based on the data released by Shenzhen Power supply Bureau in 2021. Among them, the average monthly electricity consumption in July–August was 11.541 billion kilowatts, accounting for 10.46% of Shenzhen's 2021 annual electricity consumption. In this paper, it is estimated that the annual cost savings of JZJ, JYT, JFP and JXW insulation for the air conditioning of the building model are 39,714.15 yuan, 36,244.36 yuan, 41,111.19 yuan and 41,466.06 yuan, respectively compared with the WBW working conditions without thermal insulation measures, and the effect of JXW group is the best.

When calculating the economic payback period, the discount rate of previous achievements is 5%, 10%,^56^. Limited by space, this paper only takes JXW group mortar as an example to explain the calculation process: the total area of exterior wall and roof of the typical DesignBuilder building model designed in this case is about 1506.64 m^2^ (excluding the area of doors and windows), and the thickness of all insulation layer is set to 50 mm. After calculation, the cost quota of the optimal proportion of mortar for basalt fiber thermal insulation is 409.12 yuan/m^3^, equivalent to 20.46 yuan/m^2^ (409.12 × 0.05). The labor cost is calculated according to 30 yuan/m^2^. Then the investment cost of using JXW group mortar as insulation layer in the whole building is about 76,019.03 yuan, and then the economic recovery period of JXW group mortar is calculated according to formula (3) and formula (4). The calculation results of each group of mortar are shown in Table 10.

**Table 10:** Economic analysis of TIM applications for the whole year.

Material category	JXW-01	JDZ-01	JDZ-02	JDZ-03
Energy savings (kW h)	43,684.704	41,804.398	38,172.12	43,274.89
Cost of investment (yuan)	76,019.03	70,134.03	95,701.02	73,567.01
Annual savings (yuan)	41,466.06	39,714.15	36,244.36	41,111.19
SPP	1.83	1.77	2.64	1.79
r = 5%DPP (year)	2.21	2.16	3.23	2.17
r = 6%DPP (year)	2.34	2.26	3.40	2.24
r = 7%DPP (year)	2.45	2.35	3.52	2.32
r = 8%DPP (year)	2.58	2.50	3.72	2.45
r = 9%DPP (year)	2.73	2.64	3.96	2.59
r = 10%DPP (year)	2.91	2.81	4.21	2.74

The change of data shows that the best proportion of basalt fiber thermal insulation mortar has excellent fire resistance, strength and thermal insulation properties, but due to the increase of comprehensive price caused by the addition of basalt fiber, the economic recovery period of JZJ group and JFP group mortar is shorter than that of JXW group. Although the economic recovery period of JXW group mortar is not the shortest, it is certainly better than the ordinary thermal insulation mortar on the market. Without considering the time value of capital, developers can recover the initial investment cost within two years by using JXW mortar as external wall thermal insulation material. Even if the time value of capital is taken into account, the investment cost of insulation can be recovered within three years. In addition, it is worth noting that, compared with alternative thermal insulation and energy storage materials in the construction field, the economic payback period is five years or even ten years^52–56^, and inorganic fireproof thermal insulation mortar has obvious economic advantages.

## Conclusions

The research object of this paper is thermal insulation mortar. The focus and innovation is to study the fire resistance and economy of the mortar. The ultimate goal of the research is to prepare a better performance and low-cost thermal insulation mortar based on the existing ratio. Our team adopts the research method of making samples in room and test parameters, selects basalt fiber as the admixture, makes the mortar specimen according to the standard process and tests its mechanical parameters, fire resistance performance, and finally analyzes its economic payback period according to the cost of raw materials. The main conclusions obtained in this paper are as follows:

1. In the improvement test, the bond performance of the mortar was significantly improved after the fiber was mixed, and the defoaming rate of the foam was significantly accelerated. Basalt fiber admixture in the interval of [0, 1.5%] can improve the compressive strength and bond strength of mortar, when the admixture is greater than 1.5%, it will have a negative effect on the compressive strength and the rate of bond strength growth slows down; the admixture of basalt fiber into the insulation mortar will improve the thermal conductivity and dry density of mortar. The best mixture of basalt is 1.5%, and the best quality ratio of each material is about cement: aggregate: water: dispersible latex powder: fly ash: diabase rock powder: AOS foaming agent: gelatin: basalt fiber = 1:1.67:2:0.045:0.15:0.225:0.035:0.02:0.02, and this mortar ratio is the preferable ratio of basalt fiber improvement.
2. In the refractory test, the cementitious material will inevitably deteriorate and be damaged after high temperature calcination, and the specific parameters are reduced in quality and strength; however, basalt fiber is incorporated to improve the crack resistance, compressive strength, cohesion and integrity of the mortar. Mortar with 1.5% basalt fiber admixture, after calcination under the same high temperature conditions, the burn loss rate decreased by about 10.98%, compared to the compressive strength increase of about 9.71%. Basalt fibers can significantly alleviate the adverse effects of high temperature deterioration, which is helpful for the fire performance of insulation mortar applied to building exterior walls.
3. With the help of DesignBuilder simulation software, the economic analysis of the preferred new basalt fiber insulation mortar and the finished products already applied in the market, the preferred insulation mortar not only has better performance, but also has obvious advantages in improving air comfort, reducing air conditioning energy consumption, lowering material cost and economic payback period, etc., which proves its excellent economic feasibility and It proves that it has excellent economic feasibility and engineering application prospects.

## Data Availability

Data will be made available on request. Data supporting the results of this study can be obtained by contacting the first author, Chen Ding. When sending an e-mail inquiry, be sure to state a valid reason and be brief and concise.
